# Application of Gamification Teaching in Disaster Education: Scoping Review

**DOI:** 10.2196/64939

**Published:** 2024-12-11

**Authors:** Shiyi Bai, Huijuan Zeng, Qianmei Zhong, Yuqi Shen, Lulu Cao, Mei He

**Affiliations:** 1 Department of Nursing, Mianyang Central Hospital, Affiliated with the School of Medicine, University of Electronic Science and Technology of China, No.12 Changjia Alley, Jingzhong Street, Fucheng District, Mianyang, 621000, China, 86 13778440262

**Keywords:** disaster education, disaster, gamification teaching, scoping review, gamification

## Abstract

**Background:**

With climate change, the number of natural disasters is increasing globally, and the resulting weather-related events lead to increased loss of life and property. Meanwhile, the significance of disaster education is becoming increasingly important. Despite natural disasters being hard to predict, people’s responses to such events can be improved by education and training. Gamification, an innovative teaching method, has demonstrated great potential across various fields, including disaster education.

**Objective:**

We aimed to investigate the different application types of gamification in disaster education, focusing on nursing staff, medical professionals, university students, and disaster relief workers. Specifically, the goal was to identify the types of gamified teaching used in disaster education.

**Methods:**

This scoping review was conducted according to the Joanna Briggs Institute methodology. The Participants, Concept, Context (PCC) model was used to frame the inclusion criteria. We performed a systematic search of the relevant literature across the Cochrane Library, PubMed, CINAHL, Embase, Web of Science, CNKI, Wanfang, VIPC, and SinoMed databases. Articles published in Chinese and English were selected for the review. The search was conducted to identify literature published from the establishment of the respective databases to April 21, 2024. Two researchers independently screened the literature according to the inclusion and exclusion criteria and extracted the data.

**Results:**

We included a total of 16 studies in this review, originating from 8 different countries. These studies involved 1744 participants: nursing students (n=451), medical students from other majors (n=420), college students (n=287), hospital decision makers (n=264), hospital medical staff (n=262), and disaster relief workers (n=60). The gamification approaches for teaching and learning encompassed the following 7 categories: tabletop games, serious games, scenario simulation games, virtual reality and mobile games, theme games, board games, and escape room games. The objectives of the studies were diverse. Three studies conducted randomized controlled trials, with only 1 performing a comparative analysis between different games. Two studies carried out long-term outcome evaluations.

**Conclusions:**

This scoping review explored 7 types of games for disaster education and provided evidence for future education and training. Further research is needed to establish a long-term evaluation mechanism and a better game-based teaching program to provide more insights into the future of disaster education.

## Introduction

### Background

The changing climate has increased extreme weather events, rendering global natural disasters more pronounced in terms of frequency, intensity, and complexity. At the World Climate Adaptation Summit 2021, UN Secretary-General Guterres highlighted that over the past 50 years, weather, climate, and water-related disasters have led to more than 110,000 incidents, resulting in economic losses totaling up to US $3.6 trillion [1]. Various disasters present an unparalleled challenge to the sustainable development of human societies. Comprehensive solutions should be applied immediately to reduce economic loss and guarantee the healthy development of human societies [2]. Disaster education and training can help people partly solve this problem by heightening disaster awareness and preparedness, thereby strengthening their resilience to potential threats. Hence, disaster education is important in the face of a global disaster crisis.

One study has noted that traditional disaster education methods typically involve lectures or simulation exercises [3]. In recent years, gamification teaching has gained increasing attention as an innovative and interactive learning approach that has shown great potential in various fields, such as driving skills training and medical equipment operation [4]. This method effectively reduces the cost and time of large-scale teaching by creating teaching aids and gamified software that simulate real-life scenarios and help beginners learn skills faster. Gamification is simply defined as “the use of game design elements in non-game environments to motivate learners by increasing participation, granting autonomy, and allowing learners to demonstrate competence, in line with self-determination theory” [5-12]. Research has shown that gamification in medical education has become popular due to its ability to enhance cognitive abilities such as analytical thinking, spatial reasoning, and memory retention [3]. Additionally, gamification is very beneficial for learning knowledge [13].

Existing research has explored disaster education and training for medical students, health care professionals, and university students and found that most studies have incorporated gamified teaching, which is undoubtedly a positive development. However, these studies often focus on specific groups or types of games, and a systematic understanding of the application types and effectiveness of gamified teaching in disaster education has not yet been discussed thoughtfully. Given the limitations of the current research status, especially the lack of a comprehensive literature review on the application types of gamification in disaster education, this scoping review is particularly important and urgent.

This review aims to collect and deeply analyze existing literature by summarizing various types of games that have been adopted in disaster education. Through this effort, we hope to provide educators with a comprehensive perspective for understanding the current application status of gamification in disaster education.

### Objective

The main objective of this scoping review is to map the different application types and implementation of gamification in disaster education. Thus, this review aims to conduct a statistical analysis of recent research, which focuses on application types of gamification teaching in disaster education, for nursing staff, medical professionals, university students, and disaster relief workers. Textbox 1 presents the review objective and questions from scoping review protocols.

Textbox 1. **Review objectives and questions from scoping review protocols.**

**Objectives**

Identify the types of gamified teaching used in disaster education.

**Review questions**

What types of games are used in disaster education?

**Participants**

Nursing staff, medical professionals, university students, and disaster relief workers.

**Concept**

Game types for disaster education.

**Context**

Educational or clinical environments in any geographical location.

## Methods

### Design

The utilization of scoping reviews, which is the preferred approach for synthesizing knowledge on the nature and scope of the available evidence, may not be appropriate for a more targeted and systematic review of the evidence due to its inclusiveness [14]. Scoping reviews can be employed to elucidate fundamental concepts and pinpoint knowledge gaps in emerging areas of information [15]. As there is currently no review discussing the types of gamification used in disaster education, we aimed to perform a scoping review to answer our research objectives. This review was conducted using the rigorous procedures of the Joanna Briggs Institute (JBI) methodology [16]. The report adhered to the PRISMA-ScR (Preferred Reporting Items for Systematic Reviews and Meta-Analyses Extension for Scoping Reviews; Checklist 1) [17].

### Literature Search

The system conducted a comprehensive search of relevant literature in reputable databases including the Cochrane Library, PubMed, CINAHL, Embase, Web of Science, CNKI, Wanfang, VIPC, and SinoMed databases. The selection of databases, keywords, and relevant indexing (eg, Medical Subject Headings [MeSH] and other database-specific search techniques) were finalized in collaboration with the experienced librarian. The full search strategy is presented in Multimedia Appendix 1. The search was conducted to identify literature published from the establishment of the respective databases to April 21, 2024.

### Inclusion and Exclusion Criteria

This study utilized the 2020 JBI Australia’s updated scoping review guidelines as a methodological framework [14]. The inclusion criteria were determined based on the principle of Participants, Concept, Context (PCC; Table 1). Specifically, the study focused on (1) participants, nursing staff, medical professionals, university students, and disaster relief workers; (2) concept, which was game-based instructional technology interventions provided in various types of disaster teaching; (3) context, which included game-based technology interventions in schools, hospitals, and training institutions; (4) and literature type, which was original research, including quantitative, qualitative, and mixed studies. Some sources of evidence, such as letters, conference abstracts, and news, were excluded because they would not be appropriate or useful to answer the research question.

**Table 1.** Inclusion and exclusion criteria.

Item	Inclusion criteria	Exclusion criteria
Participant	Nursing services Medical professionals College Students Disaster relief workers	All other professions
Context	Types of application of gamification	Not related to types of application of gamification in disaster education
Concept	Disaster education	Not related to disaster education
Type of studies	Original studies	Conference, abstracts, books, letters, news, etc
Language	English, Chinese	Language issue

### Study Selection

The literature was imported into EndNote20 (Clarivate) for organization and deduplication [18]. Two professionally trained researchers defined the inclusion and exclusion criteria and initially screened titles and abstracts for primary selection. Full-text reviews for secondary screening were conducted independently by one researcher, and any discrepancies were resolved through discussion with a third party. Consensus for the inclusion of articles was required from all researchers. Information extracted was tailored to the research question, including author, year, participants, sample size, concepts, design/methodology, and key findings.

### Data Charting

Before formally extracting the data, we completed two steps. First, after discussions among the research group, a data extraction table was formed according to the research purpose, which was adapted from the JBI scoping review method [14]. Second, the form was pilot-tested by SB and HZ on three randomly selected articles to ensure consistency. As the percentage of agreement was over 90% for each pair, we proceeded with data abstraction for the remaining articles and resolved any conflicts through discussion. We extracted data on the study’s first author, publication year, country of origin, participants, context, concept, design/methodology, and key findings.

### Data Synthesis

After conducting search and selection processes, graphs and tables were utilized to organize and summarize the study data. First, the extracted data were analyzed using descriptive statistics based on the characteristics of the study to report study characteristics, participants, concepts, and findings. The similarities and differences were then compared between the different studies based on the extracted data. Second, the Kirkpatrick model was used to evaluate the effectiveness of using games in disaster education. The Kirkpatrick model for evaluating training programs outlines four levels of evaluation [19]. The first level assesses trainee satisfaction with the training experience. The second level measures the trainee’s acquisition of knowledge, skills, or experience resulting from the training. The third level evaluates whether the trainee applies what they have learned (behavioral change). The last level focuses on outcomes to determine if the training positively impacts patient outcomes [20]. The data in the evidence table were collected based on the discussed topics. It is possible to synthesize any data related to the type of gamified instruction used in disaster education.

## Results

### Selection Process

**Figure 1.**
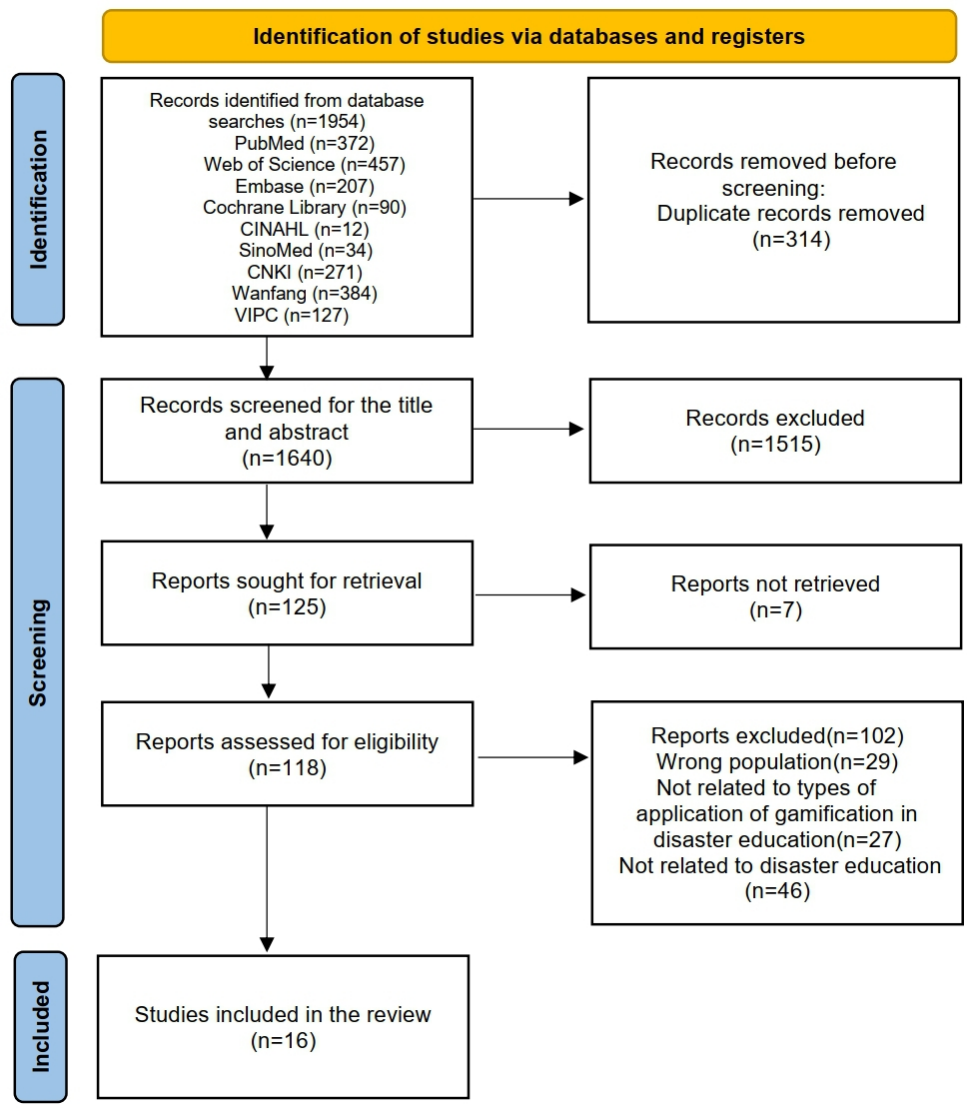
Literature screening process.

A total of 1954 abstracts were sourced from the 9 databases. First, the duplicates were removed, and 1640 records were retained. Subsequently, 1515 documents were excluded based on title and abstract screening. Full-text reviews were then conducted, which narrowed down the selection to 125 documents. Finally, 102 articles were removed following the study inclusion and exclusion criteria, and 16 articles were left for the final review [21-36]. The study search and selection process is shown in Figure 1.

### Study Characteristics

The author(s) name, participants, context, concept, design/methodology, and key findings of the included studies are summarized in Table 2. The examined articles were published between 2010 and 2024. Out of 16 studies, the majority of them (n=6) were conducted in China [23-25,27,28,36]. The remaining studies were conducted in the United States (n=2) [32,35], Germany (n=2) [22,34], Iran (n=2) [29,31], Spain (n=1) [21], Korea (n=1) [30], the United Kingdom (n=1) [26], and Malaysia (n=1) [33]. In total, 6 studies employed a pretest and posttest design [21,23,30-33]: 3 studies used randomized controlled trials [26,31,36]; 2 studies utilized a pretest, posttest, and final test design [24,25]; and 1 study adopted a single-group design [35]. The rest of the studies presented education or training process reports [22,27,34,36]. Across the 16 studies, participants included nursing students (n=451), medical students from other majors (n=420), college students (n=287), hospital decision makers (n=264), hospital medical staff (n=262), and disaster relief workers (n=60). The number of participants per study ranged from 45 to 264, resulting in a total of 1744 participants for this scoping review.

**Table 2.** Basic characteristics of the included literature [21-36].

Authors	Participants	Context	Concept	Design/Methodology	Key findings
Castro Delgado et al[Bibr R21] [21]	Fifth-year medical students (n=108)	In class	Tabletop games	Knowledge pretest and posttest	Useful for medical studies and high knowledge retention
Achatz et al [22]	Hospital decision makers (n=264)	In class	Tabletop games	Process report on training activities	Positive participants and good evaluation
Wang et al [Bibr R23][23]	Emergency medical staff (n=97)	In the emergency department	Tabletop games	Pretest and posttest	An effective method for disaster evacuation
Hu et al [24]	Nursing students (n=167)	In the HELP and RESCUE curriculum	VR-MGBA^a^	Pretest, posttest, and final test	VR-MGBAs outperformed traditional lectures in disaster evacuation
Hu et al [25]	Third-year medical students (n=131)	In disaster medicine optional course	VR-MGBA	Pretest, posttest, and final test	An effective practice tool for medical students to care for patients during natural disaster
Knight et al [26]	Doctors and nurses (n=91)	In major incident medical management and support courses	Serious games	Pragmatic controlled trial	Serious game outperforms traditional methods in enhancing learning and improving performance
Gao et al [27]	Graduates majoring in risk and disaster-related fields (n=107)	In counterfactual scenarios	Serious games	Questionnaire survey, participant observation, and interviews	Innovative serious games help to make disaster-reducing decisions
Tsai et al [28]	Students (n=67)	In-flood disaster education class	Serious games	Posttest and questionnaire evaluation	Improved student disaster prevention skills, learning interest, self-awareness, and civic responsibility
Masoumian Hosseini et al [29]	Third-year nursing students (n=60)	In class	Theme games	A pretest and posttest quasi-experimental study	An effective method for nursing students to improve their knowledge and skills of crisis management
Choi [Bibr R30]and Song [30]	Disaster relief workers (n=60)	In class	Simulation games	A single-blinded trial	Improved disaster relief worker skills, self-efficacy, and problem-solving
Masoumian Hosseini et al [Bibr R31][31]	Nursing students (n=120)	In class	Scenario simulation games	Pretest and posttest	Enhanced learning sustainability
Gue et al [32]	Medical students and emergency medicine residents (n=68)	In emergency department	Scenario simulation games	Cross-sectional prospective study, pretest, and posttest	Improved learner knowledge and confidence in managing real mass casualty incidents
Ma et al [Bibr R36][36]	Sophomore nursing students (n=104)	In class	Theme games	A randomized controlled trial	Improves nursing student disaster nursing competence than scenario simulation
Chew et al [33]	Medical students (n=113)	In class	Board games	Pretest and Posttest	A potential tool for instructional activities
Drees et al [34]	Doctors and nurses (n=74)	In class	Board games	Process report on training activities	A high acceptance method for disaster medical education
Novak et al [35]	College students (n=113)	In class	Escape room games	Single group testing	A potential method to increase student knowledge of disaster preparedness

a VR-MGBA: virtual reality mobile game–based app.

### Types of Gamification Instruction

All 16 studies presented either subjective or objective findings. Table 3 lists the types of gamified teaching and the number of articles for each type.

**Table 3.** Types of gamification.

Gamification teaching type	Number of literature	Reference
Tabletop games	3	[21-23]
Serious games	3	[25,26]
Scenario simulation game	3	[30-32]
VR-MGBA^a^	2	[24,25]
Theme games	2	[29,36]
Board games	2	[33,34]
Escape room games	1	[35]

a VR-MGBA: virtual reality mobile game-based app.

#### Tabletop Games

Three studies have applied tabletop games to disaster education practice and skill enhancement [21-23]. As a study found, for fifth-year undergraduates majoring in public health and preventive medicine, the use of tabletop games in large-scale casualty events has a very high knowledge retention rate, and students believe that this method is very useful for medical research. For hospital decision makers, using tabletop games in triage management can improve their triage and treatment speed. Integrating tabletop games into disaster education for emergency department medical staff can enhance their sense of presence and realism, as well as improve their collaboration. This reflects the transformation of disaster education from theory to practice, as well as the educational philosophy of enhancing disaster response capabilities through practice.

#### Serious Games

Three studies have employed serious games to enhance disaster education awareness and develop decision-making abilities [26-28]. For college students and medical undergraduates, serious games can enhance their disaster awareness and sense of civic responsibility as well as help them learn how to make effective decisions to reduce disaster risks. A study suggests that compared to traditional teaching, using serious games in disaster education can improve the decision-making ability of medical staff in large-scale casualty events [26].

#### Scenario Simulation Games

Three studies have applied scenario simulation games to enhance practical skills and coping abilities in disaster education [30-32]. Different categories of staff (eg, disaster relief personnel, emergency resident physicians, and nursing students) can conduct practical exercises in simulated disaster environments, enhancing their disaster response capabilities, confidence in managing complex situations, and performance in disaster. This method emphasizes enhancing practical skills and coping abilities through simulating real scenarios, which is very important for disaster education. However, researchers also pointed out that gamification can improve cognitive load and student performance, but it may increase extraneous cognitive load [37]. Therefore, scenario simulation games should not be considered stand-alone teaching methods, and games contribute to learning when used in conjunction with instruction [31].

#### Virtual Reality Mobile Game-Based Apps

Two studies have used virtual reality mobile game-based apps (VR-MGBAs) in disaster education technology to innovate and explore new teaching modes [24,25]. By combining VR-MGBAs with disaster education, a study was conducted to evaluate the effectiveness of this teaching method [25]. VR-MGBAs provide an immersive learning experience for nursing students, becoming an effective tool for learning disaster medicine, especially patient surge management. Moreover, the effectiveness of this teaching model has also been proven to be superior to traditional lectures in disaster evacuation management education and training. These studies demonstrate the potential of technological innovation in disaster education and provide useful references for the innovation and exploration of disaster education models.

#### Theme Games

Two studies have applied theme games to optimize teaching methods and evaluate their effectiveness in disaster education [29,36]. One of the studies evaluated the effectiveness of different teaching methods (theme games and scenario simulations) in improving the disaster nursing abilities of nursing students [36]. The results indicate that using theme games for teaching can effectively improve behavioral fluency and ability in crisis management, and it is even more effective than scenario simulation to some extent. This reflects the importance of optimizing teaching methods in disaster education, that is, by constantly exploring and comparing different teaching methods, we can find the most suitable teaching mode for students’ needs and learning outcomes, thereby improving the overall quality of disaster education.

#### Board Games

Two studies have employed board games to enhance the interactivity of disaster education [33,34]. Researchers have found that this form of game has a positive effect on enhancing the participation and learning outcomes of medical staff and students. For medical staff, board games are considered very suitable for disaster education because they can convey complex disaster response knowledge in a relaxed and interesting way. For medical students, board games can enhance their sense of participation in disaster classrooms, making the learning process more vivid. This reflects the concept of integrating education with entertainment, which increases the fun and interactivity of learning through gamified teaching methods, thereby enhancing students’ interest and enthusiasm for learning.

#### Escape Room Games

A study has used escape room games for specific disaster categories in disaster education [35]. For college students, escape room games in earthquake disaster teaching can enhance students’ disaster preparedness knowledge.

## Discussion

### Principal Results

From the establishment of the database until April 21, 2024, 16 studies and 7 different types of games were identified, highlighting the current lack of research on the application of gamified teaching in disaster education.

A total of 3 articles utilized tabletop games for instruction [21-23], 3 used situational simulation games [30-32], and 3 studies used serious games for teaching [26-28]. The remaining studies covered VR-MGBAs, theme games, board games, and escape room games [24,25,29,33-36]. The diverse purposes of the studies resulted in varied content designs and evaluation metrics. For instance, tabletop games teaching in mass casualty incident (MCI) response scenarios enhances knowledge retention and skill acquisition related to MCI response [21]. Situational simulation games boost learners’ confidence in managing real MCIs [32]. VR-MGBAs are effective in disaster medicine education and training, particularly for evacuation management scenarios [24,25]. Scenario-based simulation games improve disaster response competencies, including response-ability and knowledge [30]. Theme game instruction is more effective than scenario-based simulation in enhancing nursing students’ disaster response competencies [36]. The escape room games intervention has the potential to increase college students’ knowledge of disaster preparedness [35]. Additionally, board games were as effective as tabletop games in promoting interactive participation, suggesting their potential as an adjunct to instructional activities [34].

These studies collectively demonstrate the potential of gamified teaching in disaster education, emphasizing the importance of enhancing students’ practical skills and coping abilities through practice and simulation of real-life scenarios. In addition, these studies also indicate that by continuously exploring and comparing different teaching methods, the most suitable teaching mode for students’ needs and learning outcomes can be found, thereby improving the overall quality of disaster education. Researchers have also highlighted some challenges of gamified teaching in disaster education, such as the excessive number of students per group; the significant investment in resources, time, and energy; the limitations of the game scene; and its low potential for dissemination [21,22,27,32,36].

### Establishment of a Long-Term Evaluation Mechanism

The 16 literature pieces reviewed in this study vary in their description of the Kirkpatrick model levels. Four studies focused on immediate participant responses to gamification instruction [22,27,28,36], in line with the first level of the Kirkpatrick evaluation model. These studies showed that participants generally expressed high satisfaction with the gamification disaster education experience, indicating their acceptance and enjoyment of this type of instruction. Six studies utilized a pretest-posttest design [21,23,30-33], in line with the second level of the Kirkpatrick evaluation model. Additionally, only 2 studies utilized a pretest-posttest–final test design [24,25] to evaluate long-term effects, including 1 month after the posttest and the final test (6 weeks), in line with the fourth level of the Kirkpatrick evaluation model. These results indicate that future research should focus on the long-term effects of gamification instruction in disaster education. In summary, future research should comprehensively apply the Kirkpatrick model [19], assessing not only participant satisfaction and short-term learning outcomes but also behavioral change and long-term effects to ensure the effectiveness and sustainability of gamification instruction in disaster education. This will optimize gamification teaching strategies and make them more useful in disaster education.

### Comparison of Different Games

Gamification teaching methods are widely utilized in disaster education, including tabletop games, VR-MGBAs, serious games, themed games, scenario-based simulations, escape rooms, and board games, each offering unique benefits. These findings align with previous studies on game-based education. For example, VR-MGBAs provide immersive experiences [38], serious games drive learning through stories [39], while scenario simulations and escape rooms simulate real-life situations, enhancing students’ practical abilities [40]. However, existing studies have primarily focused on a single type of game, with only one study utilizing a randomized design experiment to compare the effectiveness of situational simulation games and thematic games in enhancing nursing students’ disaster-coping skills [30]. The rest of the studies did not compare these game approaches. Therefore, future research should focus on conducting a systematic comparative analysis of these game approaches to reveal differences in their actual effectiveness in disaster education and provide more evidence for the use of gamification in disaster education in the future.

### Impact on Disaster Education

All of the included studies indicate that the application of various types of games significantly improves learners’ retention of knowledge, ability to cooperate, sense of presence, realism, awareness of disasters, decision-making ability, practical skills, and coping ability in disaster education. This demonstrates the effectiveness and potential of gamified learning in disaster education. From traditional tabletop games to modern virtual reality technology, various forms of gamified teaching methods not only enrich the teaching process but also enhance students’ interest and enthusiasm for learning. This diversity and innovation provide novel ideas and directions for disaster education. Although gamified learning has shown significant advantages in disaster education, research suggests that it should be combined with other teaching methods to achieve better learning outcomes. For instance, scenario simulation games can be integrated with lectures, and virtual reality mobile games can be combined with group discussions or case studies. This diversified teaching approach can fully leverage the advantages of different teaching methods and provide students with a comprehensive and in-depth learning experience.

Finally, it is worth noting that the majority (12/16, 76%) of identified intervention studies did not have a control group, which makes it challenging to draw clear conclusions about the effectiveness of various gamified teaching methods.

### Limitations

Despite this study’s strengths, some of its limitations must be acknowledged. First, its scope was limited to peer-reviewed literature, excluding gray literature and non-original research omitted for practical reasons. In addition, only 5 English databases and 4 Chinese databases were searched, and 7 articles were not accessible in full text due to payment reasons, which may lead to missing relevant research results published in other databases. However, we have made efforts to minimize this limitation by using comprehensive search strings and utilizing literature-sharing platforms. For future reviews, a more comprehensive approach should be taken to assess more outcomes that may not be included in publications. Second, the quality of the included literature was not assessed, which precludes any conclusions on the effectiveness of gamification instruction. Third, language constraints also limited the search to Chinese and English literature, potentially resulting in relevant sources being missed. The purpose of this study was to provide a broad overview of the existing literature on the types of games used in disaster education, which can be a precursor to a systematic review. Future studies can narrow their focus to enable the use of the meta-analysis method. Despite these limitations, this review provides insight into the types of games used in disaster education that may be useful for future disaster education.

### Conclusion

This scoping review explores 7 game types used in disaster education and provides evidence for future disaster education and training, which will help to improve the ability and knowledge of nursing staff, medical professionals, university students, and disaster relief workers to cope with different types of disaster situations. Further research is needed to determine the evaluation of the long-term effectiveness of games in disaster education, conduct comparative analyses between different games, and develop more accurate training programs for more insights into future disaster education.

## Supplementary material

10.2196/64939 Multimedia Appendix 1 Search strategy.

10.2196/64939 Checklist 1 PRISMA (Preferred Reporting Items for Systematic Reviews and Meta-Analyses Extension for Scoping Reviews) checklist.

## Abbreviations

JBI: Joanna Briggs Institute
MCI: mass casualty incident
MeSH: Medical Subject Headings
PCC: Participants, Concept, Context
PRISMA-ScR: Preferred Reporting Items for Systematic Reviews and Meta-Analyses Extension for Scoping Reviews
VR-MGBA: virtual reality mobile game-based app
