# Supplementary material for: Application of Gamification Teaching in Disaster Education: Scoping Review
Source: JMIR Serious Games. 2024 Dec 11;12:e64939. doi: 10.2196/64939 (PMC11813186; doi:10.2196/64939)
Supplement: Multimedia Appendix 1 [file games-v12-e64939-s001.docx]

# **Search Terms Protocol**

**Including**

- Participants: Nursing staff, Medical professionals, University students, Disaster relief workers
- Game-based instructional technology interventions provided in various types of disaster teaching
- Game, game-based learning, serious games, gamification the context in which the game-based instructional interventions were provided, including game-based technology interventions in schools, hospitals, and training institutions
- Original research, including quantitative, qualitative, and mixed studies.
- Education
- Projects
- Journals
- English language or Chinese

**Excluding**

- Inaccessible full text
- Not related to types of application of gamification in disaster education
- Not related to disaster education
- Duplicated literature
- Literature reporting little information and poorly described data
- Research proposals conference abstracts
- Dissertations
- Guidelines
- News
- Books
- Opinion
- Policy papers,
- Letter

**PubMed**

| Number | Search Query | Results |
| --- | --- | --- |
| #1 | disasters[Title/Abstract] | 17006 |
| #2 | catastrophe[Title/Abstract] | 6081 |
| #3 | disaster[Title/Abstract] | 29123 |
| #4 | "Disasters"[Mesh] | 113685 |
| #5 | #1 OR #2 OR #3 OR #4 | 137295 |
| #6 | games[Title/Abstract] | 23411 |
| #7 | game-based learning[Title/Abstract] | 374 |
| #8 | Gamification[Title/Abstract] | 1879 |
| #9 | game[Title/Abstract] | 39058 |
| #10 | Educational game[Title/Abstract] | 237 |
| #11 | learning game [Title/Abstract] | 61 |
| #12 | Gam*[Title/Abstract] | 2509 |
| #13 | "Gamification"[Mesh] | 139 |
| #14 | #6 OR #7 OR #8 OR #9 OR #10 OR #11 OR #12 OR #13 | 56649 |
| #15 | #5 AND #14 | 372 |

**Filters: Chinese, English**

**Web of Science**

| Number | Search Query | Results |
| --- | --- | --- |
| #1 | TS=(Disasters) | 115339 |
| #2 | AB=(disaster) | 94573 |
| #3 | AB=(catastrophe) | 12748 |
| #4 | TS=(Gamification) | 8329 |
| #5 | AB=(game-based learning) | 3549 |
| #6 | AB=(Gamification) | 5636 |
| #7 | AB=(game) | 203173 |
| #8 | AB=(Educational game) | 7447 |
| #9 | AB=(learning game) | 32436 |
| #10 | #1 OR #2 OR #3 | 126424 |
| #11 | #4 OR #6 OR #5 OR #7 OR #8 OR #9 | 207437 |
| #12 | #10 AND #11 | 457 |

**Filters: Chinese, English**

**Embase**

| Number | Search Query | Results |
| --- | --- | --- |
| #1 | 'disaster'/exp | 70130 |
| #2 | disaster:ab,ti OR catastrophe:ab,ti OR 'catastrophic accident':ab,ti OR disasters:ab,ti | 43385 |
| #3 | #1 OR #2 | 78858 |
| #4 | 'game'/exp | 10422 |
| #5 | 'game model':ab,ti OR 'game theory':ab,ti OR 'games, experimental':ab,ti OR 'model, game':ab,ti OR game:ab,ti | 40947 |
| #6 | #4 OR #5 | 44773 |
| #7 | #3 AND #6 | 207 |

**Filters: Chinese, English**

**Cochrane Library**

| Number | Search Query | Results |
| --- | --- | --- |
| #1 | Mesh descriptor:[Games, Recreational] explode all trees | 81 |
| #2 | Mesh descriptor:[Disaster] explode all trees | 2280 |
| #3 | (Disasters):ti,ab,kw OR(Catastrophe) :ti,ab,kw OR(Disaster) :ti,ab,kw | 3666 |
| #4 | (Games):ti,ab,kw OR(game-based learning):ti,ab,kw OR(Gamification):ti,ab,kw OR(Recreational):ti,ab,kw | 14522 |
| #5 | #1 or #4 | 14522 |
| #6 | #2 or #3 | 5719 |
| #7 | #5 and #6 | 90 |

**CINAHL**

| Number | Search Query | Results |
| --- | --- | --- |
| #1 | games in education or game-based learning or educational games | 1015 |
| #2 | disasters or natural disasters or tsunamis or floods or drought or wildfire or earthquake or tornado or hurricane or snowstorm | 38949 |
| #3 | #1 AND #2 | 12 |

**SinoMed**

| Number | Search Query | Results |
| --- | --- | --- |
| #1 | 'Games and toys' [unweighted: expansion] | 2218 |
| #2 | 'Disasters' [unweighted: expansion] | 74452 |
| #3 | 'gamification' [common field: smart] OR 'games' [common field: smart] OR 'games and toys' [common field: smart] OR 'play' [common field: smart] AND 'and' [common field: smart] AND 'playthings' [common field: smart] OR 'video games' [common field: smart] OR 'video games' [common field: smart | 5435 |
| #4 | 'Disasters' [Common Fields: Intelligence] OR 'disasters' [Common Fields: Intelligence] OR 'catastrophes' [Common Fields: Intelligence] OR 'disaster' [Common Fields: Intelligence] | 81541 |
| #5 | #1 or #3 | 5535 |
| #6 | #2 or #4 | 81541 |
| #7 | #5 and #6 | 34 |

**CNKI**

| Number | Search Query | Results |
| --- | --- | --- |
| **#**1 | (SU='gamification' + 'game' + 'game education' + 'entertainment') AND (SU='disaster' + 'disaster' + 'natural disaster') | 271 |

**Wanfang**

| Number | Search Query | Results |
| --- | --- | --- |
| #1 | (Title or keywords: ((('disaster') or (('catastrophe')) or (('disaster')) or (('natural disaster'))) and (((('game')) or (('gamification')) or (('recreation')) or (('game education'))))) | 384 |

**VIPC**

| Number | Search Query | Results |
| --- | --- | --- |
| #1 | (K=Game OR K=Gamification OR K=Game Education OR K=Entertainment) AND (K=Disaster OR K=Disaster OR K=Natural Disaster) | 127 |
